# Anomalously warm temperatures are associated with increased injury deaths

**DOI:** 10.1038/s41591-019-0721-y

**Published:** 2020-01-13

**Authors:** Robbie M. Parks, James E. Bennett, Helen Tamura-Wicks, Vasilis Kontis, Ralf Toumi, Goodarz Danaei, Majid Ezzati

**Affiliations:** 1 MRC Centre for Environment and Health, Imperial College London, London, UK; 2 Department of Epidemiology and Biostatistics, School of Public Health, Imperial College London, London, UK; 3 The Earth Institute, Columbia University, New York, NY USA; 4 International Research Institute for Climate and Society, Columbia University, New York, NY USA; 5 Abdul Latif Jameel Institute for Disease and Emergency Analytics, Imperial College London, London, UK; 6 Space and Atmospheric Physics, Imperial College London, London, UK; 7 Harvard T.H. Chan School of Public Health, Boston, MA USA; 8 WHO Collaborating Centre on NCD Surveillance and Epidemiology, Imperial College London, London, UK

**Keywords:** Epidemiology, Society

## Abstract

Temperatures that deviate from the long-term local norm affect human health, and are projected to become more frequent as the global climate changes^1^. There are limited data on how such anomalies affect deaths from injuries. In the present study, we used data on mortality and temperature over 38 years (1980–2017) in the contiguous USA and formulated a Bayesian spatio-temporal model to quantify how anomalous temperatures, defined as deviations of monthly temperature from the local average monthly temperature over the entire analysis period, affect deaths from unintentional (transport, falls and drownings) and intentional (assault and suicide) injuries, by age group and sex. We found that a 1.5 °C anomalously warm year, as envisioned under the Paris Climate Agreement^2^, would be associated with an estimated 1,601 (95% credible interval 1,430–1,776) additional injury deaths. Of these additional deaths, 84% would occur in males, mostly in adolescence to middle age. These would comprise increases in deaths from drownings, transport, assault and suicide, offset partly by a decline in deaths from falls in older ages. The findings demonstrate the need for targeted interventions against injuries during periods of anomalously warm temperatures, especially as these episodes are likely to increase with global climate change.

## Abstract (web summary)

Bayesian spatio-temporal modeling of mortality from injuries in the contiguous USA shows increases in the number of deaths attributable to abnormal temperature fluctuations due to global heating.

## Main

Anomalously warm and cold weather events are an important public health concern in today’s world, and one of the key drivers for seeking adaptation measures against anthropogenic climate change^3–5^. Current assessments of the health effects of weather and climate, and by extension of global climate change, largely focus on parasitic and infectious diseases, and cardiorespiratory and other chronic diseases^3–8^. Less research has been conducted on injuries^9–12^, especially in a consistent way across injury types and demographic subgroups of the population. There are two reasons for investigating a potential role for temperature anomalies on injury mortality. First, death rates from injuries vary seasonally and the seasonality varies by age group^13,14^, which motivates investigating whether temperature contributes to their pathogenesis. Second, there are plausible behavioral and physiological pathways for a relationship between temperature and injury—for example, changes in alcohol drinking^15^, driving patterns and performance^12,16–24^, and levels of anger^25–27^—that motivate testing whether injury deaths are affected by temperature anomalies. Our aim was to evaluate how deaths from various injuries in the USA might be affected by anomalously warm temperatures that occur today and are expected to become increasingly common as a result of global climate change^1^.

**Fig. 1:**
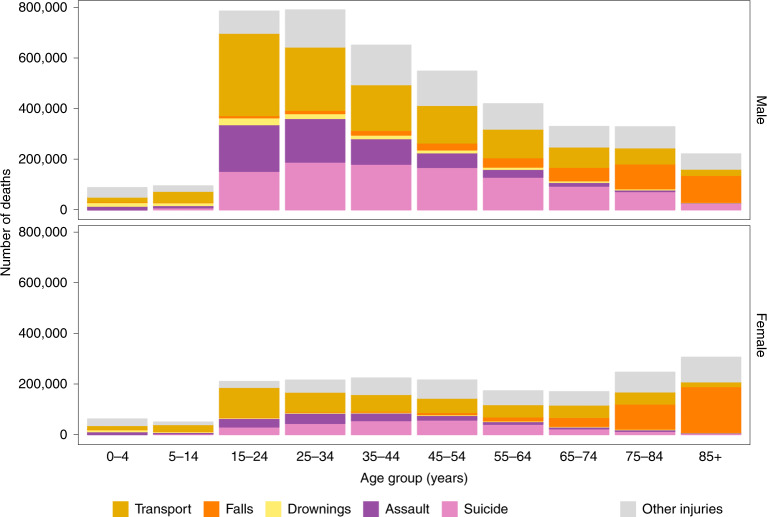
Number of injury deaths, by type of unintentional (transport, falls, drownings and other) and intentional (assault and suicide) injury, by sex and age group in the contiguous USA for 1980–2017. The top row shows the breakdown by type of injury and age group for males. The bottom row shows the breakdown by type of injury and age group for females.

We used vital registration data on all injury deaths in the contiguous USA (that is, excluding Alaska and Hawaii) from 1980 to 2017, with information on sex, age at death, underlying cause of death, and county and state of residence. From 1980 to 2017, 4,145,963 boys and men and 1,825,817 girls and women died from an injury in the contiguous USA, accounting for 9.3% and 4.2% of all male and female deaths, respectively; 95.7% of male injury deaths and 94% of female injury deaths were in those aged 15 years and older, and over half (52.3%) of male injury deaths were in those aged 15–44 years (Fig. 1). By contrast with males, there was less of an age gradient in females after age 15 years.

Injuries from transport, falls, drownings, assault and suicide accounted for 78.6% of injury deaths in males and 71.8% in females. The remainder were from a heterogeneous group of ‘other injuries’ (Fig. 1), within which the composition of injuries that led to death varied by sex and age group. Transport was the leading cause of death by injury in women younger than 75 years and in men younger than 35 years. Between ages 35 and 74 years, more men died of suicide than any other injury. Above 75 years of age, falls were the largest cause of injury-related death in both men and women.

**Fig. 2:**
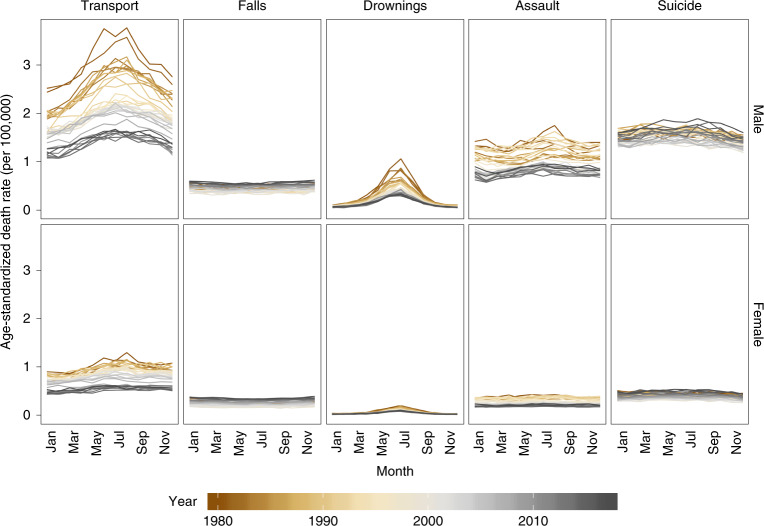
National age-standardized death rates from 1980 to 2017, by type of injury, sex and month. The top row shows the breakdown by type of injury for males. The bottom row shows the breakdown by type of injury for females.

There was a decline in age-standardized death rates of three out of five major injuries (transport, drownings and assault) from 1980 to 2017, although assault death rates have increased more recently (since 2014) (Fig. 2). By contrast, age-standardized death rates from falls increased over time whereas those from suicide initially decreased, followed by an increase to surpass 1980 levels. The largest overall decline over time was for transport deaths in both sexes and for deaths from drownings in men, which declined by more than 50% from 1980 to 2017. Age-standardized death rates for transport injuries and drownings peaked in the summer months, but deaths from other major injuries did not have clear seasonal patterns.

**Extended Data Fig. 1:**
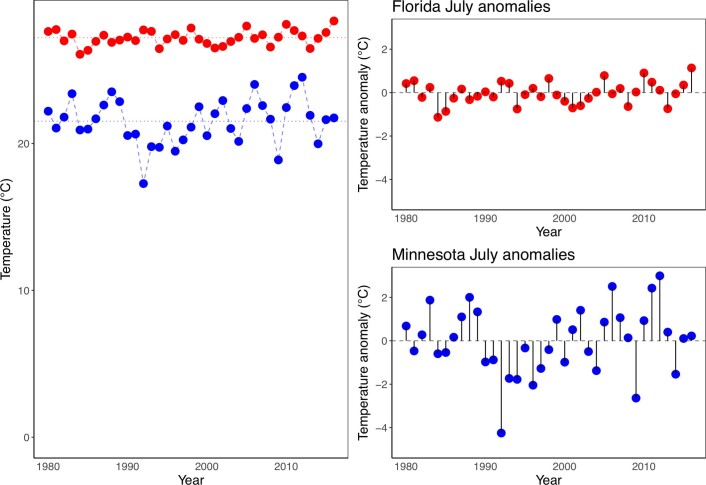
Graphic representation of temperature anomaly measure used in the analysis. The graph shows how monthly temperatures in July two example states (Florida in red and Minnesota in blue) (left panel) for 1980–2017 are used to calculate temperature anomalies. As seen, a warmer state like Florida (top right) can have a smaller inter-annual variation in a particular month (here, July) compared with a cooler state like Minnesota (bottom right).

**Extended Data Fig. 2:**
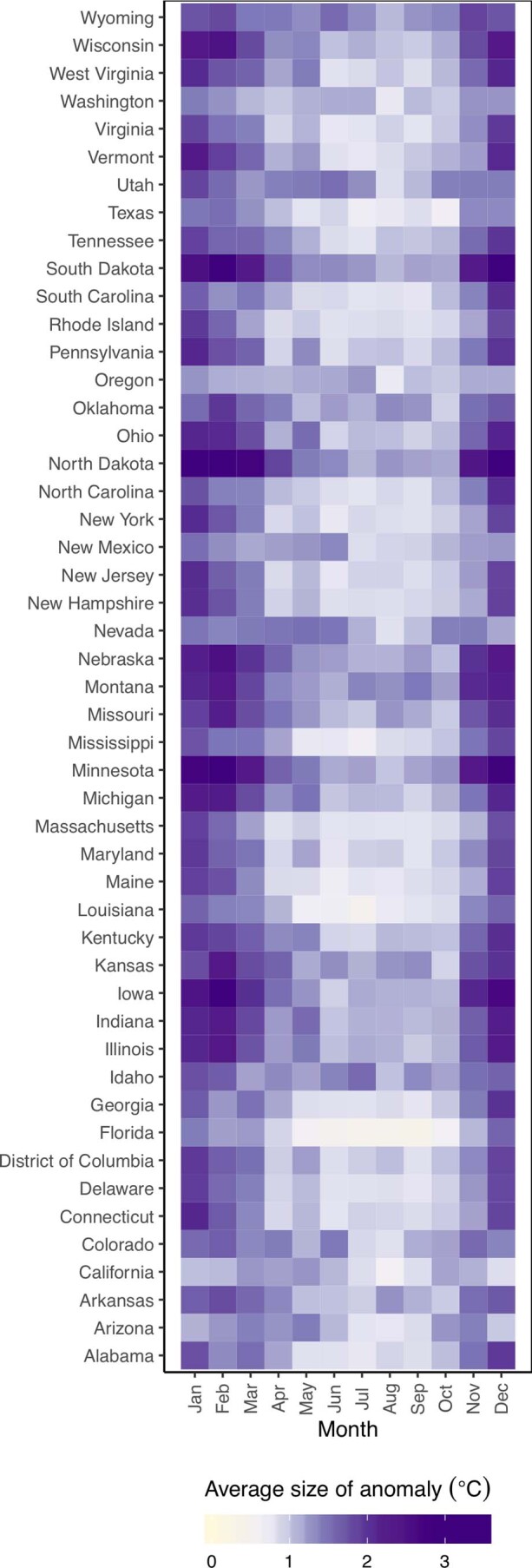
Average size of temperature anomaly (°C) from 1980 to 2017, by state and month. The value for each state and month is the mean of the absolute size of anomaly, be it cold or warm, and hence gives an indication of the scale of anomalies around the local average temperatures.

We defined a measure of anomalous temperature for each county and month, which represents the deviation from the county’s average temperature in that month over the entire analysis period (see Extended Data Fig. 1). County-level anomalies were aggregated to the state level with the use of population weights. This generated a number for each state and month that measured deviation from long-term average of the state in that month. Average size of anomaly over the study period (1980–2017), a measure of how variable temperatures are around their state–month long-term average, ranged from 0.4 °C for Florida in September to 3.4 °C for North Dakota in February (see Extended Data Fig. 2). Taken across all states and months, the average size of anomaly had a median value of 1.2 °C. Temperature anomalies were largest in January and December and smallest in August and September. In addition, they were larger in northern and central states than in southern and coastal ones.

**Fig. 3:**
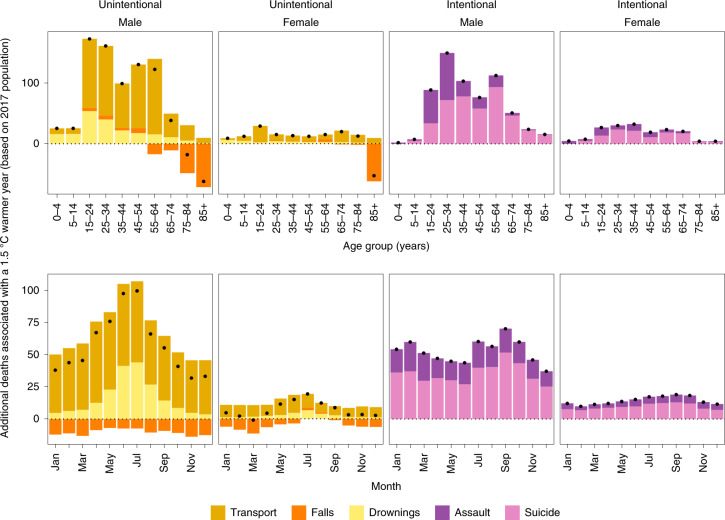
Additional annual injury deaths for the 2017 US population in the year in which each month was +1.5 °C warmer compared with 1980–2017 average temperatures. The top row shows the breakdown by type of injury, sex and age group. The bottom row shows the breakdown by type of injury, sex and month. Black dots represent net changes in deaths for each set of bars. See Extended Data Fig. 3 for results of the scenario of 2 °C warmer.

**Extended Data Fig. 3:**
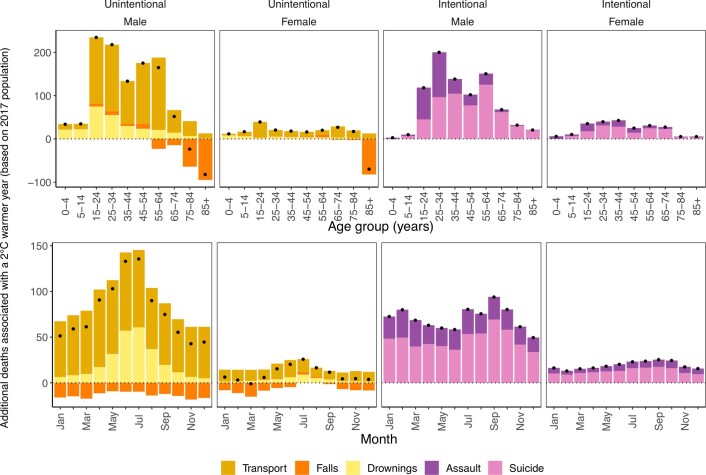
Additional annual injury deaths for the 2017 US population in year in which each month was +2 °C warmer compared with 1980–2017 average temperatures. The top row shows breakdown by type of injury, sex and age group. The bottom row shows the break down by type of injury, sex and month. Black dots represent net changes in deaths for each set of bars.

**Extended Data Fig. 4:**
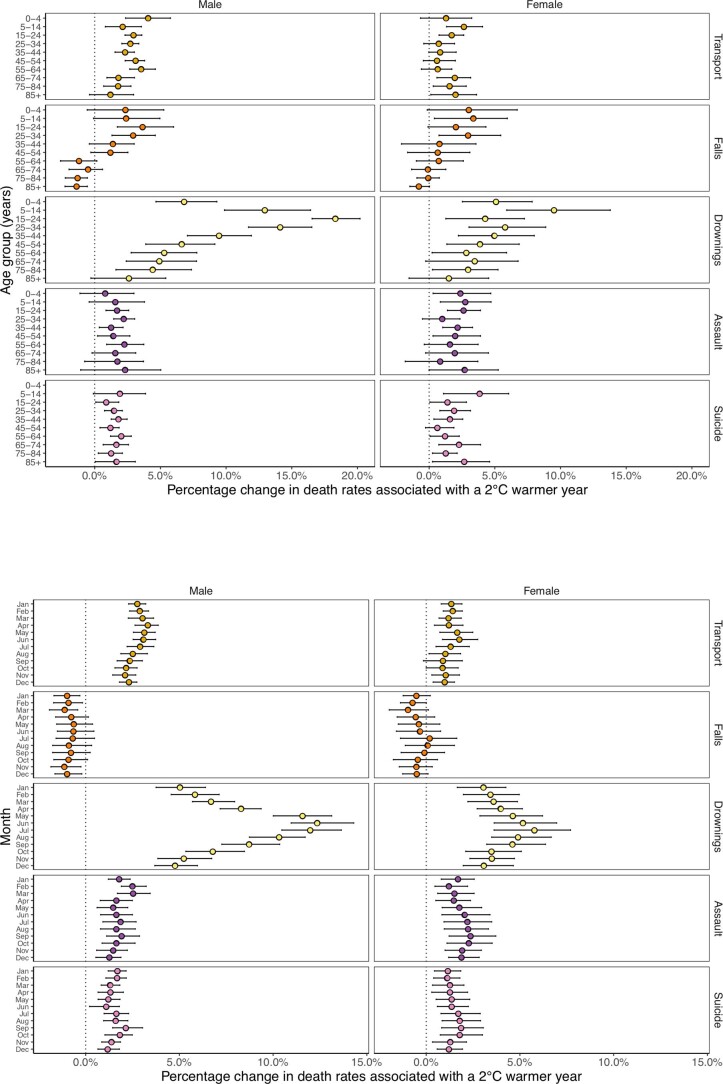
Percent change in death rates in year in which each month was +2 °C compared with 1980–2017 average temperatures by type of injury, sex and (A) age group or (B) month. Colored dots show the posterior means and error bars represent 95% credible intervals, both obtained at the posterior draw level.

We analyzed the association of monthly injury death rates with anomalous temperature using a Bayesian spatio-temporal model, described in detail in Methods. We used the resultant risk estimates, and the age- and sex-specific death rates from each injury in 2017, to estimate additional deaths if each month in each state were +1.5 °C above its long-term average, as envisioned under the Paris Climate Agreement^2^. We present additional results, based on +2 °C, which is the upper boundary of the Paris Climate Agreement, as Extended Data Figs. 3 and 4. Based on this analysis, there would be an estimated 1,601 (95% credible interval 1,430–1,776) excess injury deaths, equivalent to 0.75% of all injury deaths in 2017, in a year in which each month in each state was +1.5 °C warmer than its long-term average (Fig. 3). The number of excess injury deaths would increase to 2,135 (1,906–2,368), equivalent to 1.0% of all injury deaths in 2017, in each year in which each month in each state was +2 °C warmer than its long-term average (see Extended Data Fig. 3).

Deaths from drownings, transport, assault and suicide would increase, partly offset by a decline in deaths from falls in middle and older ages and in winter months (Fig. 3). Most excess deaths would be from transport injuries (739; 650–814 in the +1.5 °C warmer scenario) followed closely by suicide (540; 445–631). Of the excess deaths, 84% would occur in males and 16% in females. Of all male excess deaths, 92% would occur in those aged 15–64 years, who have higher rates of deaths from transport and suicide. In those aged 85 years and older, there would be an estimated decline in injury deaths, because deaths from falls are expected to decline in a warmer year.

**Fig. 4:**
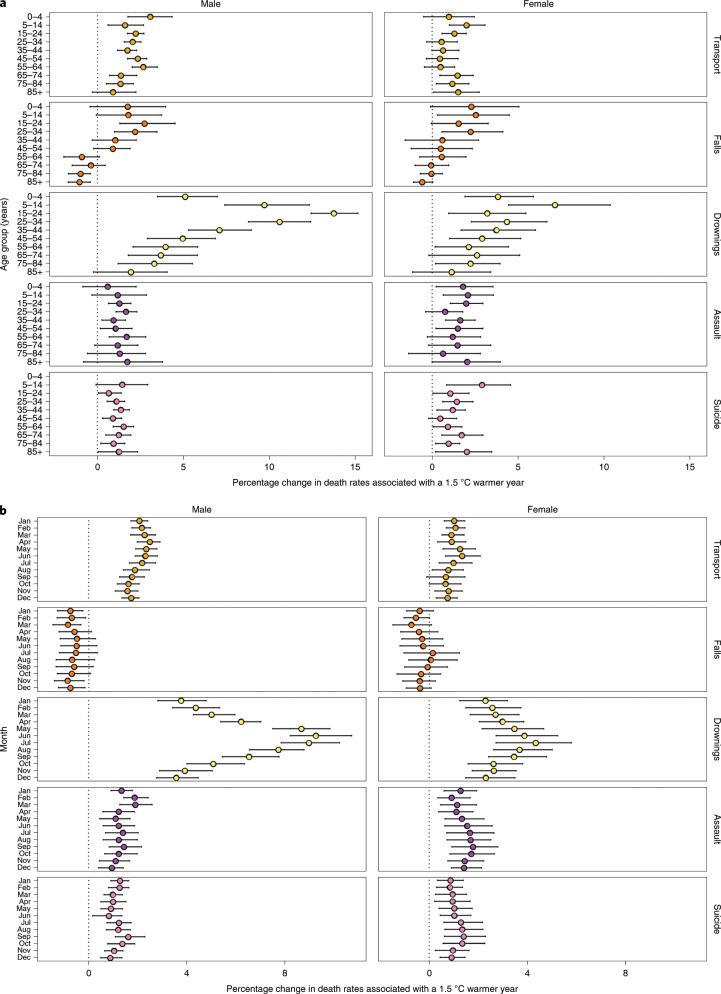
Percentage change in death rates in year in which each month was +1.5 °C compared with 1980–2017 average temperatures by type of injury, sex and age group or month. **a**, Percentage change in death rates by injury, sex and age group. **b**, Percentage change in death rates by injury, sex and month. Colored dots show the posterior means and error bars represent 95% credible intervals, both obtained at the posterior draw level. See Extended Data Fig. 4 for the scenario of 2 °C warmer.

Proportionally, deaths from drownings are estimated to increase more than those of other injury types—by as much as 13.7% (12.5, 15.2) for a +1.5 °C anomaly in men aged 15–24 years (Fig. 4). The smallest proportional increase was that of assault and suicide (less than 3% in all age and sex groups). There was a larger percentage increase in transport deaths for males than for females, especially in young and middle ages (for example, 2.0% (1.6, 2.6) for 25- to 34-year-old men versus 0.5% (−0.3, 1.4) for women of the same age) (Fig. 4). We present additional results, based on +2 °C, in Extended Data Fig. 4.

That anomalously warm temperature influences deaths from drownings, although not previously quantified, is highly plausible because swimming is likely to be more common when the temperature is higher. The higher relative and absolute impacts on men compared with women may reflect differences in their behaviors. For example, over half of swimming deaths for males occur in natural water, compared with about a quarter for females^28^. The former may rise more in warmer weather. Similarly, deaths from falls declined in older ages, but increased in young ages, because falls in elderly people are more likely to be due to slipping on ice than falls in younger people^29–31^.

The pathways from anomalous temperature to transport injury are more varied. First, driving performance deteriorates at higher temperatures^20–23^. Furthermore, alcohol consumption increases in warm temperatures^15^, which also provides an explanation for why teenagers, who are more likely than other age groups to crash while intoxicated^32^, could experience a larger proportional rise in deaths from transport, when temperatures are anomalously warm, than older adults. Last, warmer temperatures generally increase road traffic in North America^12,16–19,24^; coupled with more people outdoors in warmer weather^33^, this increase could lead to more fatal collisions.

Pathways linking anomalously high temperatures and deaths from assault and suicide are less established. One hypothesis is that more time spent outdoors in anomalously warmer temperatures leads to an increased number of face-to-face interactions, and hence arguments, confrontations and ultimately assaults^34,35^. These effects could be compounded by the greater anger levels linked to higher temperatures^25–27^. However, further research on the association of temperature and assault, and the factors mediating it, is needed^36^. Regarding suicide, it has been hypothesized that a higher temperature is associated with higher levels of distress in younger people^37^. Nevertheless, the mechanisms for the links between temperature and mental health require further investigation, including whether the relationship varies by age and sex, as indicated by our results. Future research should also investigate the extent to which the increased risk of injury death as a result of anomalous temperature depends on community characteristics, such as poverty and deprivation, social connectivity and cohesion, quality of roads and housing, public transportation options, emergency response and social services.

**Extended Data Fig. 5:**
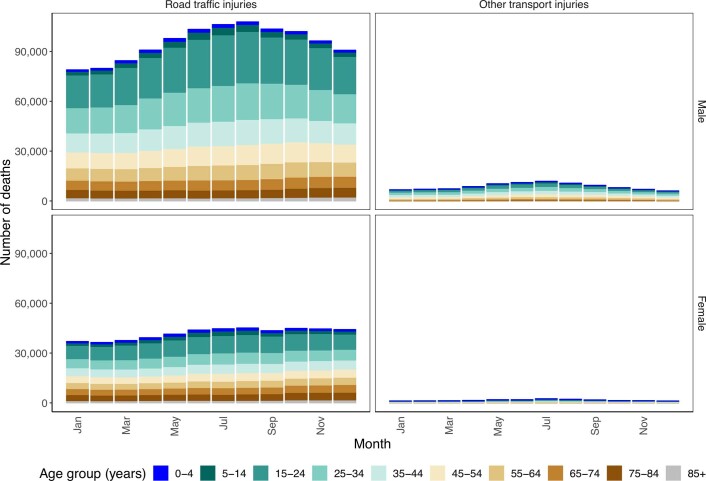
Number of deaths by type of transport injury, month, sex and age group in the contiguous United States for 1980–2017.

The major strength of our study is that we have comprehensively modeled the association of temperature anomaly with injury by type of injury, month, age group and sex. Our measure of temperature anomaly internalizes the long-term historical experience of each state, and is closer to what climate change may bring about than solely examining daily episodes, or average temperature to which people have adapted. To utilize this metric, we integrated two large disparate national datasets on mortality (vital statistics) and meteorology (ERA5), and developed a bespoke Bayesian spatio-temporal model. A limitation of our study is that, like all observation studies, we cannot rule out confounding of results due to other factors. As described above, our statistical model by design adjusts for factors related to month, state and state–month that either are invariant over time or change linearly. Rather, the confounding factors would be those with anomalies similar to those of the monthly temperature in each state, such as air pollution. However, to our knowledge, there is currently no evidence of an association between air pollution and injury mortality. We analyzed the associations between anomalous temperature and injury mortality at the state level, because the small number of events and computational demands made county-level analyses unfeasible. Analyses at finer spatial resolution, such as county or district level^38^, would be ideal because the impacts of anomalously warm and cold temperature on deaths from injuries may depend on socioeconomic (for example, poverty, social connectivity and cohesion, availability of guns), environmental (for example, availability of swimming pools, distance to bodies of water), infrastructure (for example, quality and safety of roads, public transportation options), and health and social services (for example, counseling and mental health services, emergency response). We used categories of injuries that are relevant to public health purposes and for designing and implementing interventions. It may be possible to further split each category. For example, 92% of all transport injuries in males and 96% in females are from road traffic injuries, with the remainder being classified as other transport injuries (see Extended Data Fig. 5). Similarly, suicides can be classified based on the means of suicide. To the extent that these subcategories are relevant for interventions, they should be separately analyzed in future studies. Finally, as with any Bayesian model, choices of prior distributions and hyper-parameters are necessary. There are alternatives to the priors we used. For example, our weakly informative gamma priors could have been replaced by penalized complexity priors^39^ or uniform priors on the standard deviation scale^40^. We tested a limited number of alternatives and found that our results were robust to such specifications.

Our work highlights how deaths from injuries are currently susceptible to temperature anomalies and could also be modified by rising temperatures resulting from climate change, unless countered by social infrastructure and health system interventions that mitigate these impacts. Although absolute impacts on mortality are modest, some groups, especially men who are young to middle aged, experience larger impacts than other age and sex groups. Therefore, a combination of public health interventions that broadly target injuries in these groups—for example, targeted messaging for younger males on the risks of transport injury and drowning—and those that trigger in relation to forecast high-temperature periods—for example, additional targeted blood alcohol level checks—should be a public health priority.

## Methods

### Data sources

We used data on deaths by sex, age, underlying cause of death and state of residence in the contiguous USA from 1980 to 2017 through the National Center for Health Statistics (NCHS) (https://www.cdc.gov/nchs/nvss/dvs_data_release.htm) and on the population from the NCHS bridged-race dataset for 1990–2017 (https://www.cdc.gov/nchs/nvss/bridged_race.htm) and from the US Census Bureau before 1990 (https://www.census.gov/data/tables/time-series/demo/popest/1980s-county.html). We did not include Alaska and Hawaii (which together made up 0.5% of the US population in 2017) because their climates and environment are distinct from other states due to their substantial physical distance. We calculated monthly population counts through linear interpolation, assigning each yearly count to July.

The underlying cause of death was coded according to the *International Classification of Diseases* (ICD) system (the 9th revision from 1980 to 1998 and the 10th revision thereafter). The six million injury deaths fell into six categories: transport, falls, drownings, assault, suicide and an aggregate set of other injuries (see Supplementary Table 1). We report the results of all of these categories except other injuries (1,402,941 deaths or 23% of total injury deaths during 1980–2017), because the composition of this aggregate group varies by sex, age group, state and time.

We obtained data on temperature from ERA5, which uses data from global in situ and satellite measurements to generate a worldwide meteorological dataset, with full space and time coverage over our analysis period^41^. We used gridded estimates measured four times daily at a resolution of 30 km to generate monthly temperatures by county.

### Anomalous temperature metric

With few exceptions^9,42^, current climate change risk assessments extrapolate from associations of daily mortality with daily temperature^7,8,43–45^. Climate change will, however, fundamentally modify weather, including seasonal weather patterns, compared with long-term averages, and hence can disrupt existing forms of adaptation. To mimic the conditions that may arise with global climate change, we developed methodology to examine how deviations from the long-term average temperature may impact injury death rates.

We first defined a measure of anomalous temperature for each county and month, which represents the deviation from the average temperature of the county in that month over the entire analysis period. To calculate the magnitude of temperature anomaly, we first calculated average temperatures for each month in each county over the entire 38 years of analysis. We subtracted these long-term average temperatures from respective monthly temperature values to generate a temperature anomaly time series for each month and year in each county (see Extended Data Fig. 1). The temperature anomaly metric measures the extent to which the temperature experienced in a specific month, year and county is warmer or cooler than the long-term average to which the population has acclimatized. These values can be different for different months in the same county, and different counties in the same month. Furthermore, a county with a higher, but more stable, temperature in a specific month has smaller anomalies than one with a lower, but more inter-annually variable, temperature. County-level anomalies were aggregated to the state level with the use of population weights for analyzing their associations with mortality.

### Statistical methods

We analyzed the association of monthly injury death rates with anomalous temperature using a Bayesian spatio-temporal model, which leveraged variations over space and time to infer associations. We modeled the number of deaths in each month in each year as following a Poisson distribution:

$${\mathrm{deaths}}_{\mathrm{state-time}}\sim {\mathrm{Poisson}}({\mathrm{death}}\;{\mathrm{rate}}_{\mathrm{state-time}}\times {\mathrm{population}}_{\mathrm{state-time}})$$

with the log-transformed death rates modeled as a sum of components that depend on location (state) of death, month of year, overall time (in months) and temperature anomaly:

$$\begin{array}{l} \log \left( {\mathrm{death}}\;{\mathrm{rate}_{\mathrm{state - time}}} \right) = (\alpha _0 + \beta _0\times \mathrm{time}) + (\alpha _{\mathrm{state}} + \beta _{\mathrm{state}}\times \mathrm{time})\\\qquad\qquad\qquad\qquad\qquad + (\alpha _{\mathrm{month}} + \beta _{\mathrm{month}}\times \mathrm{time}) + \zeta _{\mathrm{state} - \mathrm{month}}\\\qquad\qquad\qquad\qquad\qquad + (\psi _{\mathrm{state - month}}\times \mathrm{time}) + \nu _{\mathrm{time}}\\\qquad\qquad\qquad\qquad\qquad + ({\upgamma}_{\mathrm{month}}\times \mathrm{anomaly}_{\mathrm{state - time}}) + {\upvarepsilon}_{\mathrm{state - time}} \end{array}$$

The model contained terms that represent the national level and trend in mortality, with *α*_0_ as the common intercept and *β*_0_ the common slope with overall time. Death rates also vary by month, which may be partly related to temperature and partly due to other monthly factors; monthly variations tend to be smooth across adjacent months^13^. Therefore, we allowed each month of the year to systematically have a different mortality level and trend, with *α*_month_ the month-specific intercept and *β*_month_ the month-specific slope with overall time. We used a first-order random walk prior for the monthly random intercepts and slopes, widely used to characterize smoothly varying trends^46^. The random walk had a cyclic structure, so that December was adjacent to January.

We also included state random intercepts and slopes for death rates, with *α*_state_ as the state-specific intercept and *β*_state_ the state-specific slope with overall time. These terms measure deviations of each state from national values, and allow variation in level and trend in mortality by state. We modeled the state-level random intercepts and slopes using the Besag, York and Mollie spatial model^47^, which includes both spatially structured random effects with an intrinsic conditional autoregressive prior and spatially unstructured, independent and identically distributed gaussian random effects. The extent to which information is shared between neighboring states depends on the uncertainty of death rates in a state and the empirical similarity of death rates in neighboring states. We also included state–month interactions for intercepts and slopes (*ζ*_state–month_ and *ψ*_state–month_), to allow variation in mortality levels and trends in a particular state for different months and vice versa. These state–month interactions were modeled as independent and identically distributed, and therefore were of type I space–time interactions^48^. Non-linear change over overall time (in months) was captured by a first-order random walk, *v*_time_^46^. To ensure identifiability, each set of random walk terms or state random effects was constrained to sum to zero.

Finally, we included a term that relates log-transformed death rate to the above-defined state–month temperature anomaly, *γ*_month_ × anomaly_state–time_. The coefficients of *γ*_month_ represent the logarithm of the monthly death rate ratio per 1 °C increase in anomaly. There was a separate coefficient for each month, which means that an anomaly of the same magnitude could have different associations with injury mortality in different months. As with the month-specific intercepts and trends, we used a cyclic first-order random walk to smooth the coefficient of the temperature anomaly across months. An over-dispersion term (*ε*_*s*tate–time_) captured the variation unaccounted for by other terms in the model, modeled as *N*(0,$\sigma _{\it{\epsilon }}^2$). We used weakly informative priors so that parameter estimation was driven by the data. As in previous analyses^49,50^, hyper-priors were defined on the logarithm of the precisions of the random effects, in other words on log(1/𝜎^2^). These were modeled as logGamma(𝜃, 𝜹) distributions with shape 𝜃 = 1 and rate 𝜹 = 0.001. The same hyper-priors were used for all precision parameters of the random effects in the model. For the common slope, we used *N*(0, 1,000) and for the common intercept a flat prior.

In addition to representing the spatial (across states) and temporal (across months and years) patterns of mortality, the intercept terms (*α*_month_, *α*_state_, *ζ*_state–month_) in our statistical model implicitly adjust for unobserved factors that influence mortality at the state, month and state–month level; the slope terms (*β*_month_, *β*_state_, *ψ*_state–month_) do so for changes in these factors over time^49^. This means that the only confounding factors would be those that have the same state–month anomaly as temperature.

We fitted the models using integrated nested Laplace approximation (INLA), and the R-INLA software, which is computationally more efficient than traditional Markov Chain Monte Carlo for Bayesian inference^51^. The uncertainty in our results was obtained from 5,000 draws from the posterior marginal of each month’s excess relative risk. The reported 95% credible intervals are the 2.5th to 97.5th percentiles of the sampled values.

Analyses were done separately by injury type, because different injuries can have differing associations with anomalously warm and cold temperatures. Analyses were also done separately by sex and age group (0–4 years, 10-year age groups from 5 years to 84 years, and 85+ years) because injury death rates vary by age group and sex (see Fig. 1 and Supplementary Table 2), as might their associations with temperature. We used the resultant risk estimates, and the age–sex-specific death rates from each injury in 2017, to calculate additional deaths if each month in each state was +1.5 °C above its long-term average, not only realistic in our lifetime under the current projections of global climate change, but as agreed under the Paris Climate Agreement^2,52^. This +1.5 °C rise is also within the range of the size of the anomaly experienced by some states (see Extended Data Fig. 2). For these calculations, we multiplied the actual death counts for each month, sex, state and age group in 2017 by the corresponding excess relative risk, which was calculated as the exponential of the coefficient of the temperature anomaly term from the above analysis. We did similar calculations for +2 °C, which is the upper boundary of the Paris Climate Agreement, and present these as Extended Data Figs. 3 and 4.

### Sensitivity analyses

We conducted sensitivity analyses to assess how much our results might depend on the temperature metric used to generate anomalous temperature. First, rather than building our monthly temperature anomalies based on daily mean temperatures, we used daily maxima and minima. These measures were strongly correlated to those generated from daily means (see Supplementary Table 3), and therefore we did not run models using these alternatives.

Second, together with temperature anomaly based on daily mean temperatures, we also included a second measure of anomaly in the model. We tested three different measures for this sensitivity analysis: (1) temperature anomaly calculated based on the 90th percentile (°C) of daily mean temperatures within a month, compared with the average of the 90th percentiles for each state and month; (2) number of days in a month above the long-term 90th percentile of average temperature for each state and month (adjusted for length of month); and (3) number of episodes of 3+ day episodes above the long-term 90th percentile of average temperature for each state and month (adjusted for length of month). These additional measures were related to more extreme anomalous situations, which may be relevant if the impacts on injuries are related to more extreme temperatures and their frequency in each month.

The correlations among these variables and anomaly based on the mean were between 0.60 and 0.89 (see Supplementary Table 4). The estimated rate ratios of the temperature anomaly based on daily means (that is, the anomaly measure used in the main analysis) were robust to the addition of alternative measures of anomaly, whereas the coefficients of the additional measures were generally not statistically significant and with large credible intervals. Therefore, we did not include the alternative additional measures of extreme anomalous temperature in the main analysis.

### Comparison with previous studies

Although there are no previous studies of how deviations of monthly temperature from the long-term average are associated with injury mortality, our results are broadly in agreement with both those that have analyzed associations with absolute temperature and those for specific injury types. A study of suicide in US counties over 37 years (1968–2004) estimated that a 1 °C higher monthly temperature would lead to a 0.7% rise in suicides^9^, compared with our findings of 0.7–1.5% in males and 0.5–2.9% in females at different ages for a +1.5 °C anomaly. A cross-sectional analysis in 100 US counties found that a 1 °C higher temperature would lead to a 1.3% increase in death rates from road traffic injuries^24^, compared with our finding of 0.6–3.1% in males and 0.5–2.0% in females for a +1.5 °C anomaly. In a study of six French heatwaves during 1971–2003, mortality from unintentional injuries rose by up to 4% during a heatwave period compared with a non-heatwave baseline^10^. A study of daily mortality from all injuries from Estonia found a 1.24% increase in mortality when the daily maximum temperature went from the 75th to the 99th percentile of long-term distribution^11^.

### Reporting Summary

Further information on research design is available in the Nature Research Reporting Summary linked to this article.

## Online content

Any methods, additional references, Nature Research reporting summaries, source data, extended data, supplementary information, acknowledgements, peer review information; details of author contributions and competing interests; and statements of data and code availability are available at 10.1038/s41591-019-0721-y.

## Supplementary information

Supplementary Information Supplementary Tables 1–4.

Reporting Summary

## Data Availability

ERA5 temperature data are downloadable from https://www.ecmwf.int/en/forecasts/datasets/reanalysis-datasets/era5. Vital statistics files with geographical information can be requested through submission of a proposal to the NCHS (https://www.cdc.gov/nchs/nvss/nvss-restricted-data.htm).

## Code availability

The computer code for the Bayesian model used in this work is available at http://globalenvhealth.org/code-data-download.
