# Supplementary material for: Anomalously warm temperatures are associated with increased injury deaths
Source: Nat Med. 2020 Jan 13;26(1):65–70. doi: 10.1038/s41591-019-0721-y (PMC6957467; doi:10.1038/s41591-019-0721-y)
Supplement: Supplementary file 1 — Supplementary Tables 1–4. [file 41591_2019_721_MOESM1_ESM.pdf]

nature medicine

SUPPLEMENTARY INFORMATION
https://doi.org/10.1038/s41591-019-0721-y

In the format provided by the authors and unedited.

Anomalously warm temperatures are associated with increased injury deaths

Robbie M. Parks[1,2,3,4], James E. Bennett[1,2,5], Helen Tamura-Wicks[1,2], Vasilis Kontis[1,2], Ralf Toumi[6], Goodarz Danaei[7] and Majid Ezzati[1,2,5,8]★

[1]MRC Centre for Environment and Health, Imperial College London, London, UK. [2]Department of Epidemiology and Biostatistics, School of Public Health, Imperial College London, London, UK. [3]The Earth Institute, Columbia University, New York, NY, USA. [4]International Research Institute for Climate and Society, Columbia University, New York, NY, USA. [5]Abdul Latif Jameel Institute for Disease and Emergency Analytics, Imperial College London, London, UK. [6]Space and Atmospheric Physics, Imperial College London, London, UK. [7]Harvard T.H. Chan School of Public Health, Boston, MA, USA. [8]WHO Collaborating Centre on NCD Surveillance and Epidemiology, Imperial College London, London, UK. *e-mail: majid.ezzati@imperial.ac.uk

Supplementary Table 1. Injury groups used in the analysis with ICD-9 and ICD-10 codes.

Injury type		ICD-9	ICD-10
Unintentional	Transport	E800-E849	V01-V99
	Falls	E880-E888	W00-W19
	Drownings	E910	W65-W74
	Other unintentional (not analysed)	E850-E869, E890-E909, E911-E928	W20-W64, W75-X59
Intentional	Suicide	E950-E959	X60-X84
	Assault	E960-E969	X85-Y09
Intention undetermined (not analysed)		E980-E989	Y10-Y34
Legal intervention and operations of war (not analysed)		E970-E979, E990-E999	Y35-Y36
Complications of medical and surgical care (not analysed)		E870-E879, E930-E949	Y40-Y84
Sequelae of external causes (not analysed)		E929	Y85-Y89

Supplementary Table 2. Number of deaths and population over the study period (1980-2017) for injuries included in the analysis.

Sex	Age group (years)	Transport	Falls	Drowning	Suicide	Assault	Population (millions)
Male	0-4	19,263	1,828	14,110	0	14,137	379.6
	5-14	42,669	1,324	11,158	7,748	8,974	759.5
	15-24	316,862	8,801	26,335	147,423	180,145	801.9
	25-24	243,115	12,592	18,433	183,075	168,401	806.3
	35-34	175,783	17,389	13,617	175,251	98,664	748.8
	45-44	144,482	26,760	10,941	162,956	56,557	646.6
	55-54	110,084	36,343	8,420	126,006	29,811	508.0
	65-74	78,582	51,674	6,027	91,763	14,365	342.9
	75-84	62,262	95,526	4,136	70,682	6,531	176.4
	85+	23,756	103,976	1,596	25,633	1,861	49.9
Female	0-4	15,366	1,040	7,499	0	11,357	362.7
	5-14	25,912	489	3,517	2,971	5,894	725.1
	15-24	114,825	1,372	2,773	29,346	33,585	768.3
	25-24	75,607	2,096	2,756	43,114	39,843	797.5
	35-34	64,139	3,996	2,757	53,786	29,759	759.6
	45-44	55,040	8,301	2,737	56,141	17,900	672.5
	55-54	47,243	15,337	2,443	40,004	10,302	555.5
	65-74	47,478	34,426	2,213	22,261	7,572	417.0
	75-84	46,699	96,857	2,270	12,705	6,086	266.9
	85+	18,243	176,591	1,171	4,573	2,620	112.0

Supplementary Table 3. Pearson's correlation coefficients between monthly anomalies generated from daily mean temperature and daily maximum and minimum temperatures. Each correlation coefficient was calculated in each state for each month for 1980-2017, then averaged over all states for each month.

**Month**	**Mean daily temperature and maximum daily temperature**	**Mean daily temperature and minimum daily temperature**
January	0.98	0.98
February	0.98	0.98
March	0.97	0.97
April	0.97	0.96
May	0.96	0.94
June	0.95	0.92
July	0.97	0.94
August	0.96	0.93
September	0.93	0.91
October	0.91	0.93
November	0.96	0.97
December	0.97	0.98

Supplementary Table 4. Pearson's correlation coefficients between anomaly of mean daily temperature and measures of extreme anomalous temperature described in Methods. Each correlation coefficient was calculated in each state for each month for 1980-2017, then averaged over all states for each month.

Temperature variables	Anomaly of mean (main analysis)	Anomaly of 90th percentile	Number of days above long-term 90th percentile	Number of 3+ day episodes above long-term 90th percentile
Anomaly of mean (main analysis)		0.79	0.75	0.6
Anomaly of 90th percentile	0.79		0.89	0.77
Number of days above long-term 90th percentile	0.75	0.89		0.86
Number of 3+ day episodes above long-term 90th percentile	0.6	0.77	0.86	
