# Supplementary material for: Anomalously warm temperatures are associated with increased injury deaths
Source: Nat Med. 2020 Jan 13;26(1):65–70. doi: 10.1038/s41591-019-0721-y (PMC6957467; doi:10.1038/s41591-019-0721-y)
Supplement: Supplementary file 2 — Reporting Summary [file 41591_2019_721_MOESM2_ESM.pdf]

nature research

Corresponding author(s): Majid Ezzati

Last updated by author(s): Nov 19, 2019

Reporting Summary

Nature Research wishes to improve the reproducibility of the work that we publish. This form provides structure for consistency and transparency in reporting. For further information on Nature Research policies, see Authors & Referees and the Editorial Policy Checklist.

Statistics

For all statistical analyses, confirm that the following items are present in the figure legend, table legend, main text, or Methods section.

n/a	Confirmed	
☒	☐	The exact sample size (n) for each experimental group/condition, given as a discrete number and unit of measurement
☒	☐	A statement on whether measurements were taken from distinct samples or whether the same sample was measured repeatedly
☒	☐	The statistical test(s) used AND whether they are one- or two-sided<br>*Only common tests should be described solely by name; describe more complex techniques in the Methods section.*
☒	☐	A description of all covariates tested
☒	☐	A description of any assumptions or corrections, such as tests of normality and adjustment for multiple comparisons
☐	☒	A full description of the statistical parameters including central tendency (e.g. means) or other basic estimates (e.g. regression coefficient) AND variation (e.g. standard deviation) or associated estimates of uncertainty (e.g. confidence intervals)
☒	☐	For null hypothesis testing, the test statistic (e.g. F, t, r) with confidence intervals, effect sizes, degrees of freedom and P value noted<br>*Give P values as exact values whenever suitable.*
☐	☒	For Bayesian analysis, information on the choice of priors and Markov chain Monte Carlo settings
☒	☐	For hierarchical and complex designs, identification of the appropriate level for tests and full reporting of outcomes
☒	☐	Estimates of effect sizes (e.g. Cohen's d, Pearson's r), indicating how they were calculated

Our web collection on statistics for biologists contains articles on many of the points above.

Software and code

Policy information about availability of computer code

Data collection	Processing of administrative/environmental data was conducted using the statistical software R (version 3.6.0)
Data analysis	All analyses were conducting using the statistical software R (version 3.6.0). The code will be available on http://globalenvhealth.org/code-data-download/

For manuscripts utilizing custom algorithms or software that are central to the research but not yet described in published literature, software must be made available to editors/reviewers. We strongly encourage code deposition in a community repository (e.g. GitHub). See the Nature Research guidelines for submitting code & software for further information.

Data

Policy information about availability of data

All manuscripts must include a data availability statement. This statement should provide the following information, where applicable:
- Accession codes, unique identifiers, or web links for publicly available datasets
- A list of figures that have associated raw data
- A description of any restrictions on data availability

ERA5 temperature data are downloadable from https://www.ecmwf.int/en/forecasts/datasets/reanalysis-datasets/era5. Vital statistics files with geographical information can be requested through submission of a proposal to NCHS (https://www.cdc.gov/nchs/nvss/nvss-restricted-data.htm).

Field-specific reporting

Please select the one below that is the best fit for your research. If you are not sure, read the appropriate sections before making your selection.

☐ Life sciences ☒ Behavioural & social sciences ☐ Ecological, evolutionary & environmental sciences

For a reference copy of the document with all sections, see nature.com/documents/nr-reporting-summary-flat.pdf

Behavioural & social sciences study design

All studies must disclose on these points even when the disclosure is negative.

Study description	We used data on mortality and temperature over 38 years (1980-2017) in the entire contiguous USA and formulated a Bayesian spatio-temporal model to quantify how anomalous temperatures, defined as deviations of monthly temperature from the local average monthly temperature over the entire analysis period, affect deaths from unintentional (transport, falls and drownings) and intentional (assault and suicide) injuries, by age group and sex.
Research sample	We used vital registration data on all injury deaths in the contiguous USA (i.e., excluding Alaska and Hawaii) from 1980 to 2017, with information on sex, age at death, underlying cause of death and county and state of residence.
Sampling strategy	No sampling was undertaken. All injury deaths and populations in the study area and study period were included in the analysis.
Data collection	We used data on deaths by sex, age, underlying cause of death and state of residence in the contiguous USA from 1980 to 2017 through the National Center for Health Statistics (NCHS) (https://www.cdc.gov/nchs/nvss/dvs_data_release.htm) and on population from the NCHS bridged-race dataset for 1990 to 2017 (https://www.cdc.gov/nchs/nvss/bridged_race.htm) and from the US Census Bureau prior to 1990 (https://www.census.gov/data/tables/time-series/demo/popest/1980s-county.html).
Timing	We used data collected from 1980 to 2017.
Data exclusions	We did not include Alaska and Hawaii, (which together made up 0.5% of the US population in 2017) because their climates and environment are distinct from other states due to their substantial physical distance. We also reported the results of all injury categories except other injuries (1,402,941 deaths or 23% of total injury deaths during 1980-2017), because the composition of this aggregate group varies by sex, age group, state and time.
Non-participation	n/a
Randomization	Our study is observational, and we did not carry out experiments.

Reporting for specific materials, systems and methods

We require information from authors about some types of materials, experimental systems and methods used in many studies. Here, indicate whether each material, system or method listed is relevant to your study. If you are not sure if a list item applies to your research, read the appropriate section before selecting a response.

Materials & experimental systems

n/a	Involved in the study	
☒	☐	Antibodies
☒	☐	Eukaryotic cell lines
☒	☐	Palaeontology
☒	☐	Animals and other organisms
☒	☐	Human research participants
☒	☐	Clinical data

Methods

n/a	Involved in the study	
☒	☐	ChIP-seq
☒	☐	Flow cytometry
☒	☐	MRI-based neuroimaging
